# Supplementary material for: Binding Efficacy and Thermogenic Efficiency of Pungent and Nonpungent Analogs of Capsaicin
Source: Molecules. 2018 Dec 4;23(12):3198. doi: 10.3390/molecules23123198 (PMC6321193; doi:10.3390/molecules23123198)
Supplement: Supplementary file 1 [file molecules-23-03198-s001.zip › ALL EXTRA BLOTS_SUPPLEMENTAL.pdf]

TRPV1

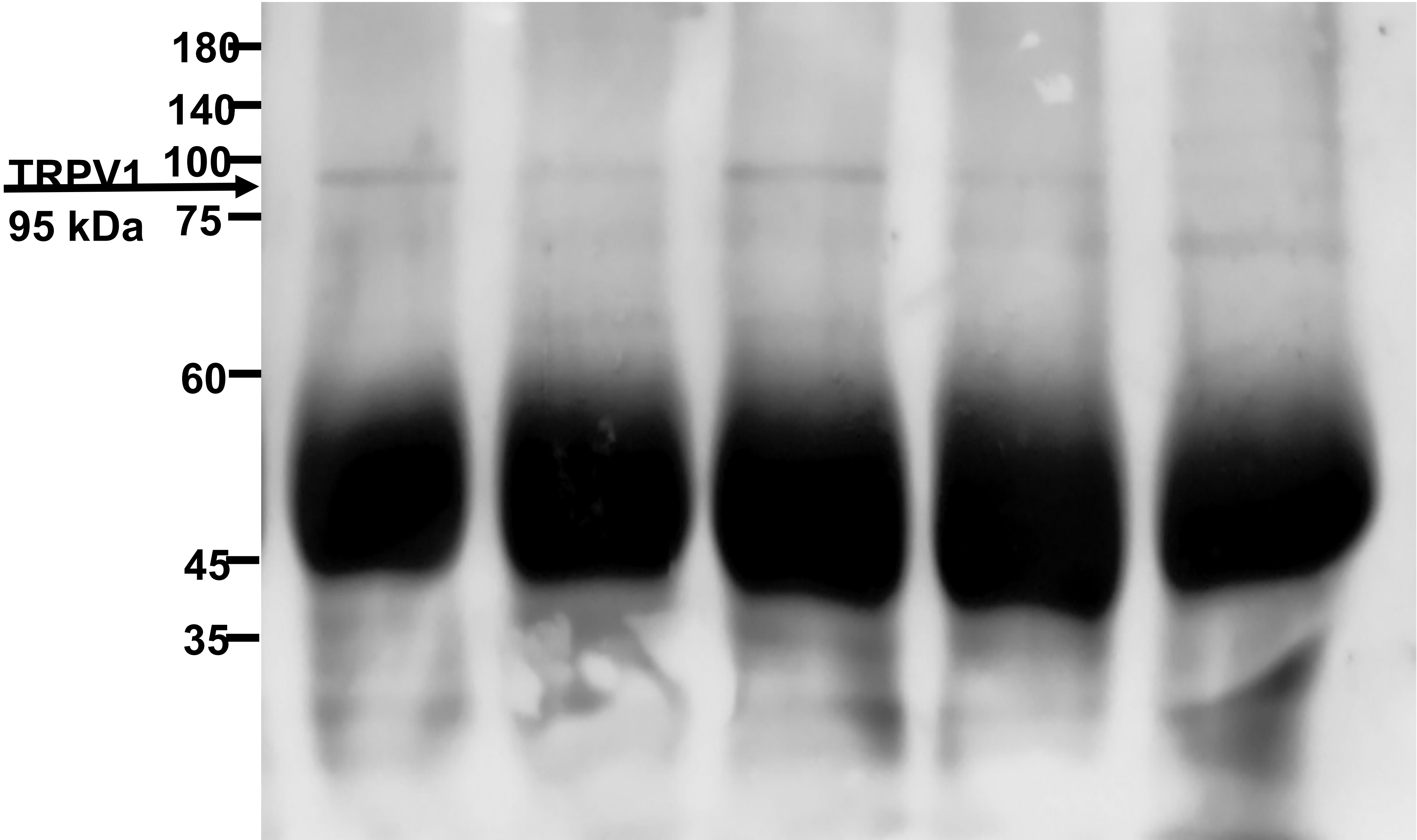

TRPV1

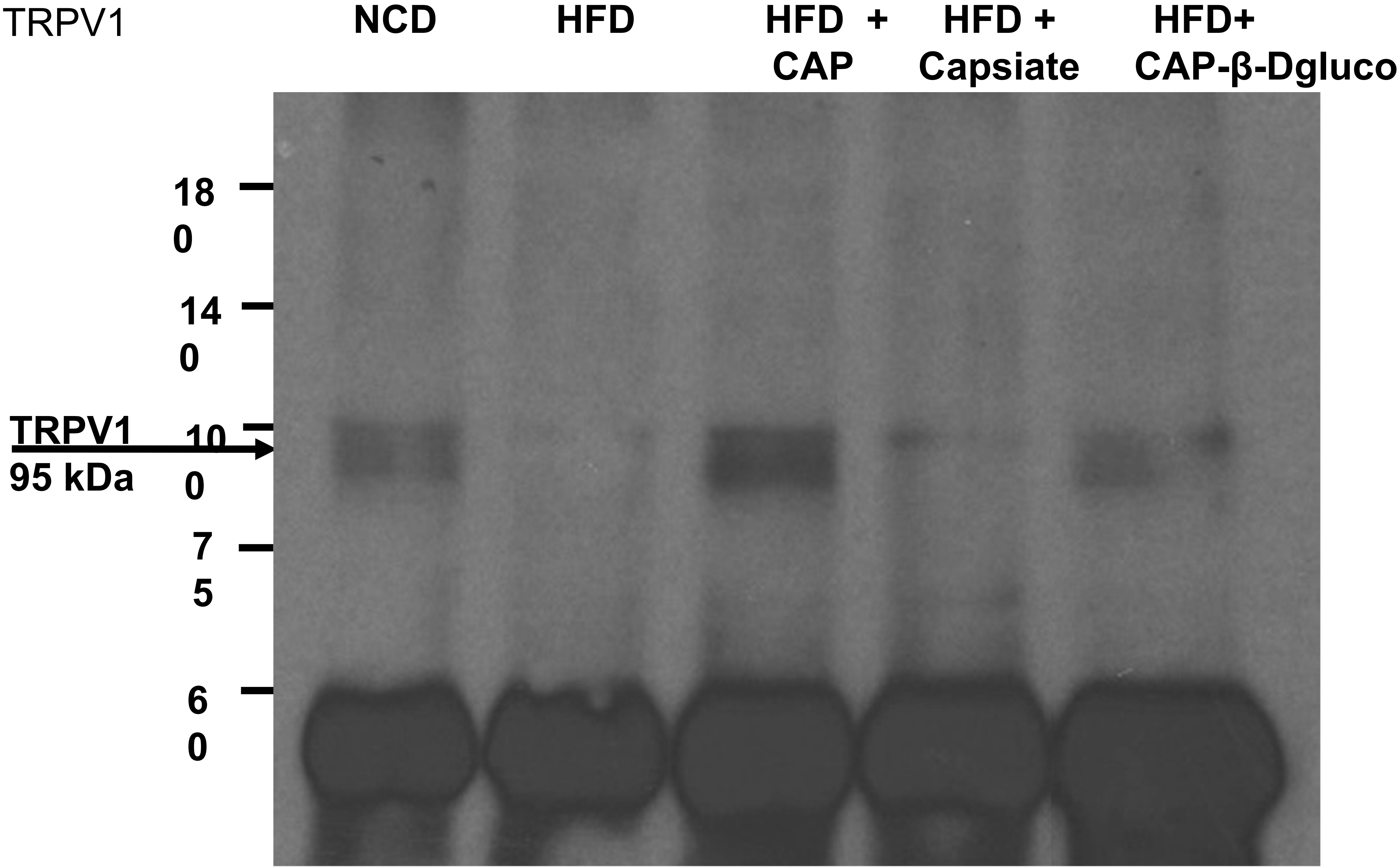
NCD
HFD
HFD + CAP
HFD + Capsiate
HFD+ CAP-β-Dgluco
180
140
100
75
60
TRPV1
95 kDa

PPAR α

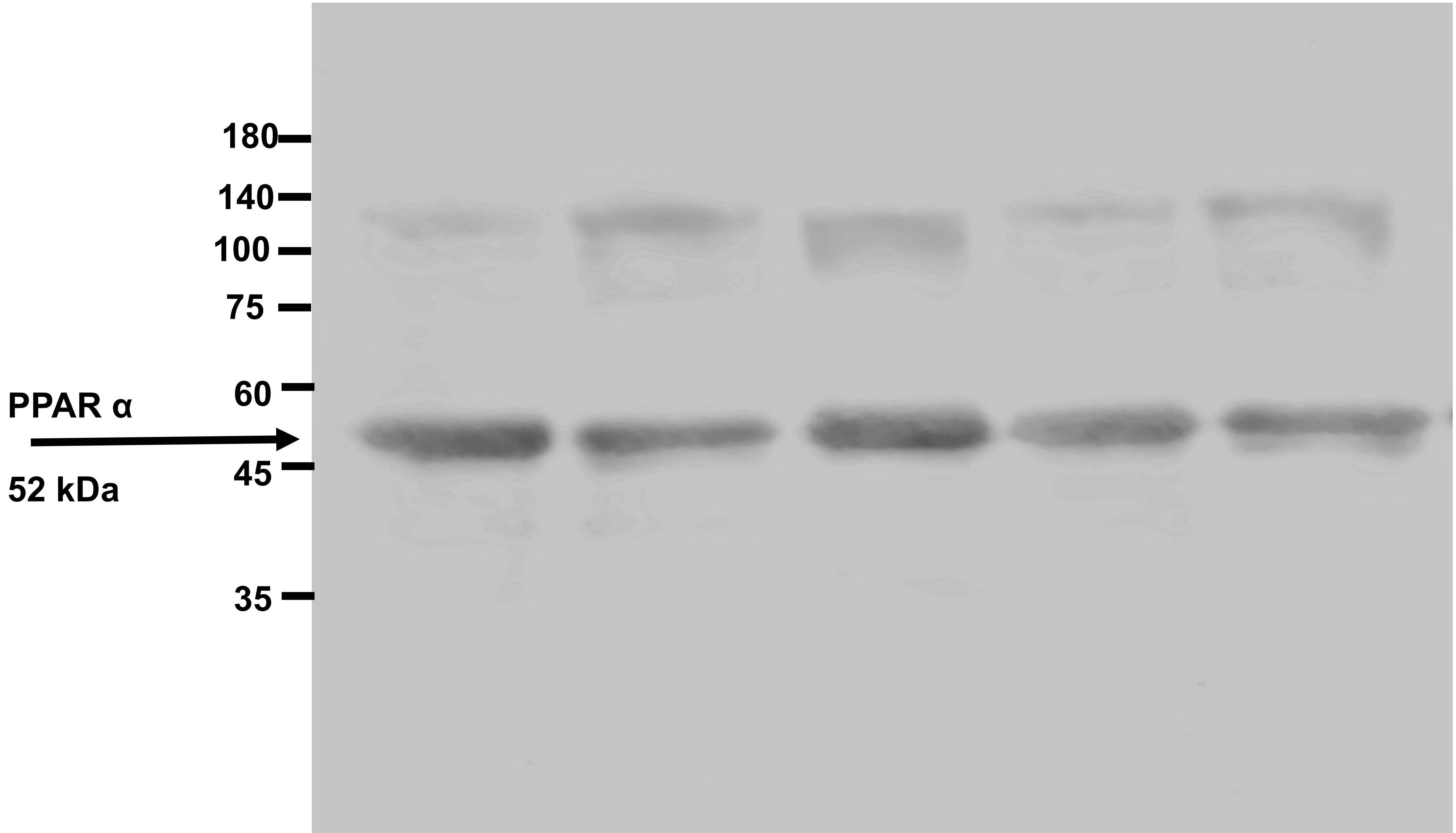

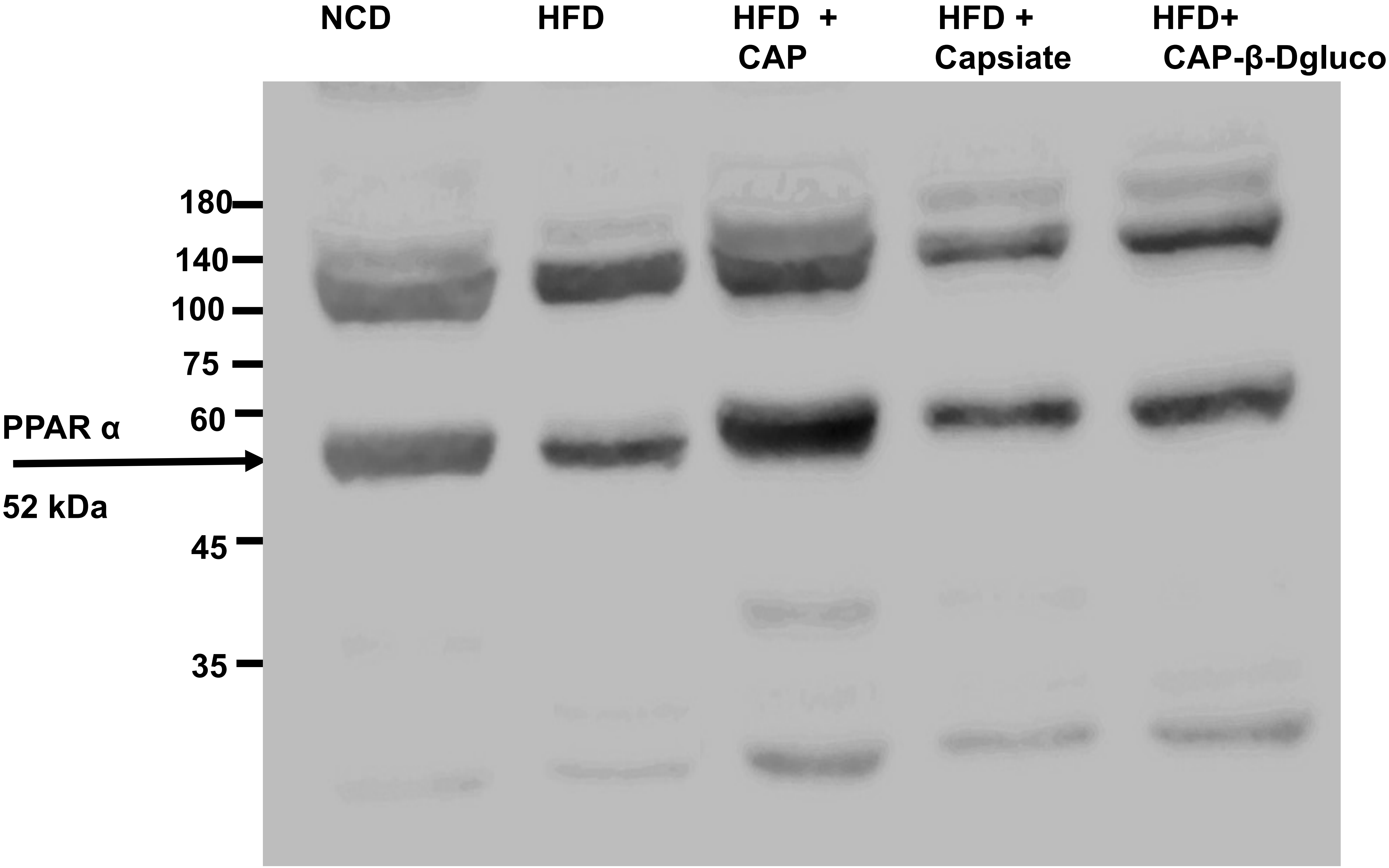
NCD
HFD
HFD + CAP
HFD + Capsiate
HFD+ CAP-β-Dgluco
180
140
100
75
60
PPAR α
52 kDa
45
35

BMP8b

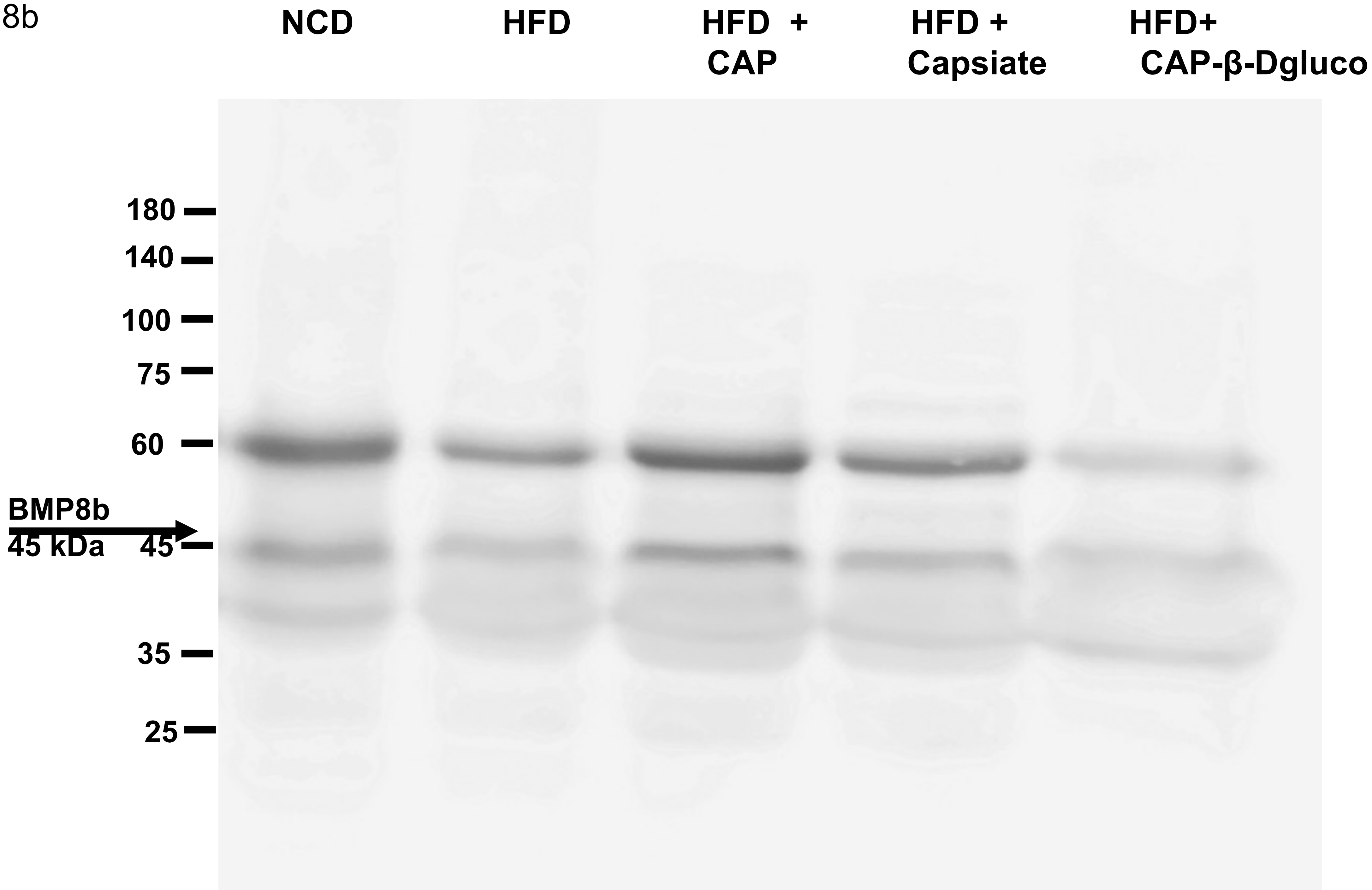
NCD
HFD
HFD + CAP
HFD + Capsiate
HFD+ CAP-β-Dgluco
180
140
100
75
60
BMP8b 45 kDa
45
35
25

BMP8b

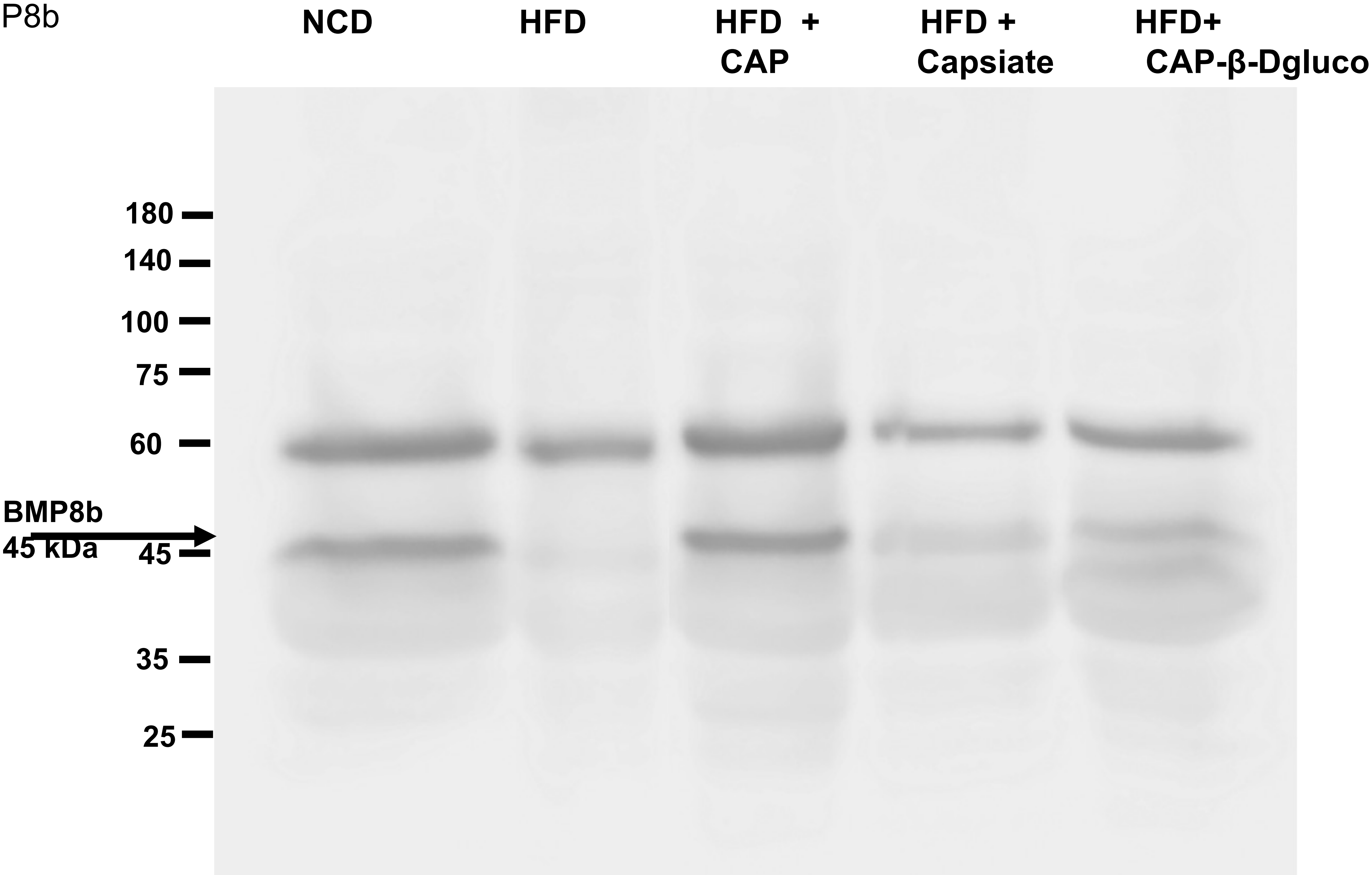

NCD
HFD
HFD + CAP
HFD + Capsiate
HFD+ CAP-β-Dgluco
180
140
100
75
60
45
35
25
BMP8b 45 kDa

UCP1

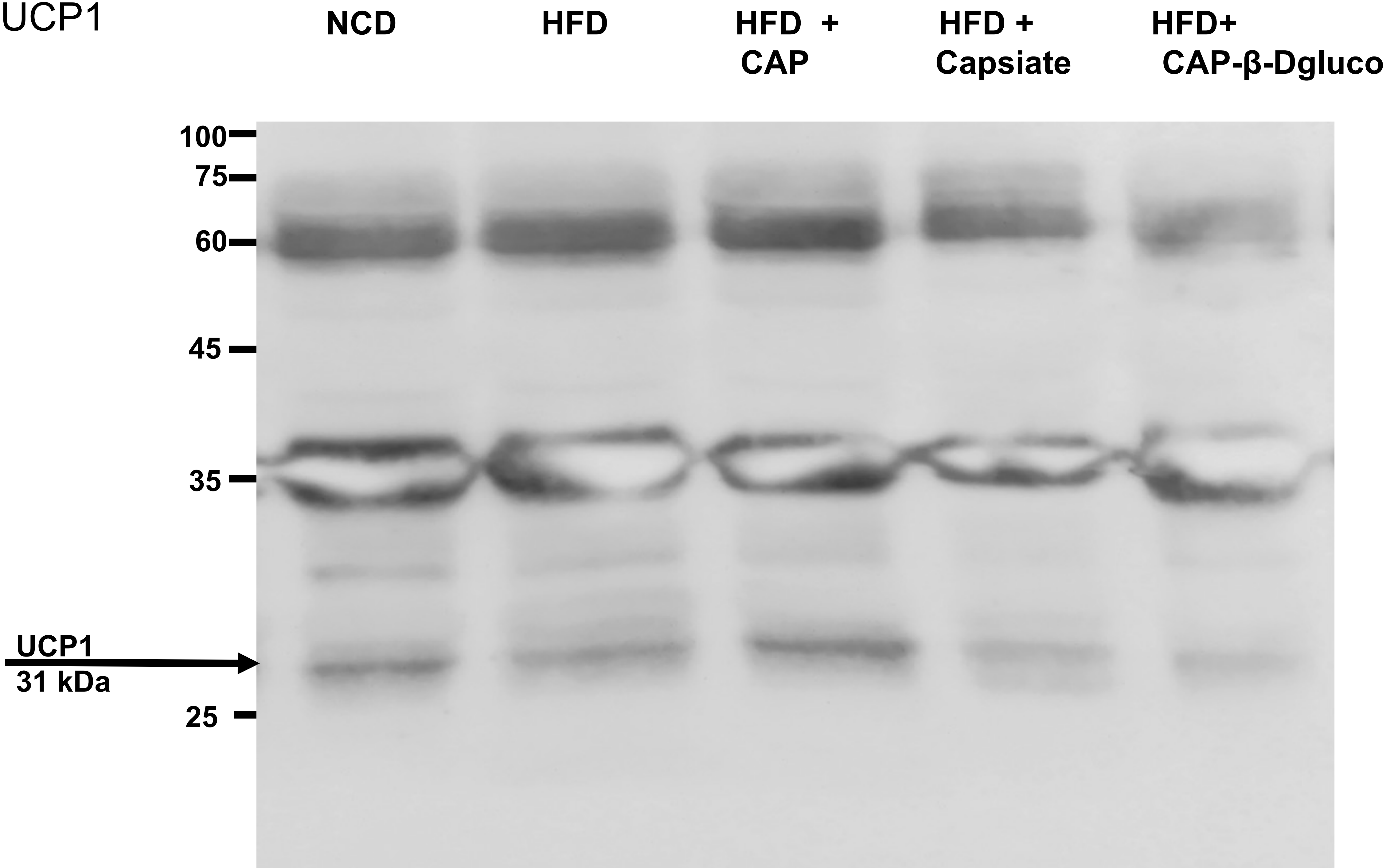
NCD
HFD
HFD + CAP
HFD + Capsiate
HFD+ CAP-β-Dgluco
100
75
60
45
35
25
UCP1
31 kDa

UCP1

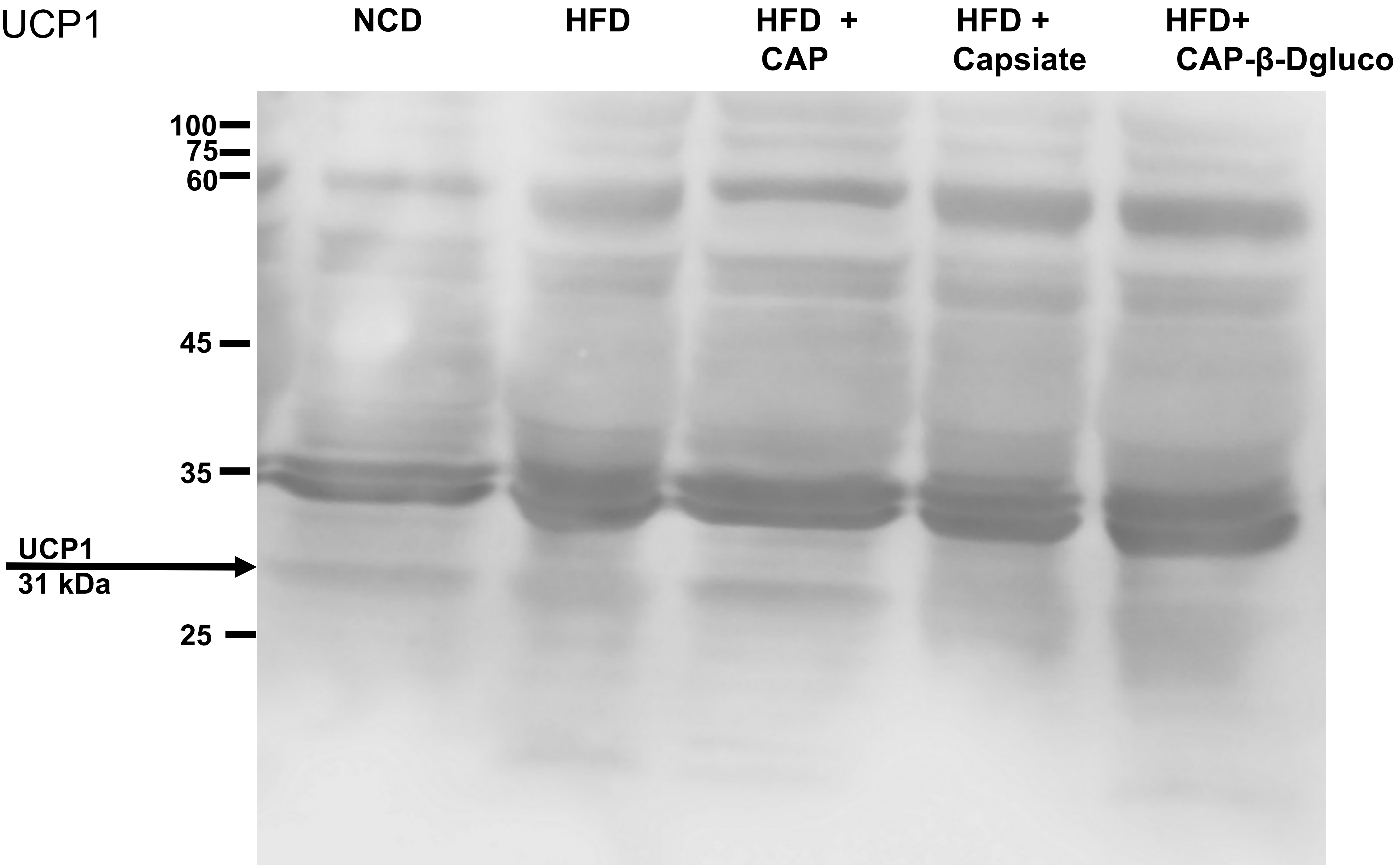

NCD
HFD
HFD + CAP
HFD + Capsiate
HFD+ CAP-β-Dgluco
100
75
60
45
35
25
UCP1 31 kDa

SIRT1

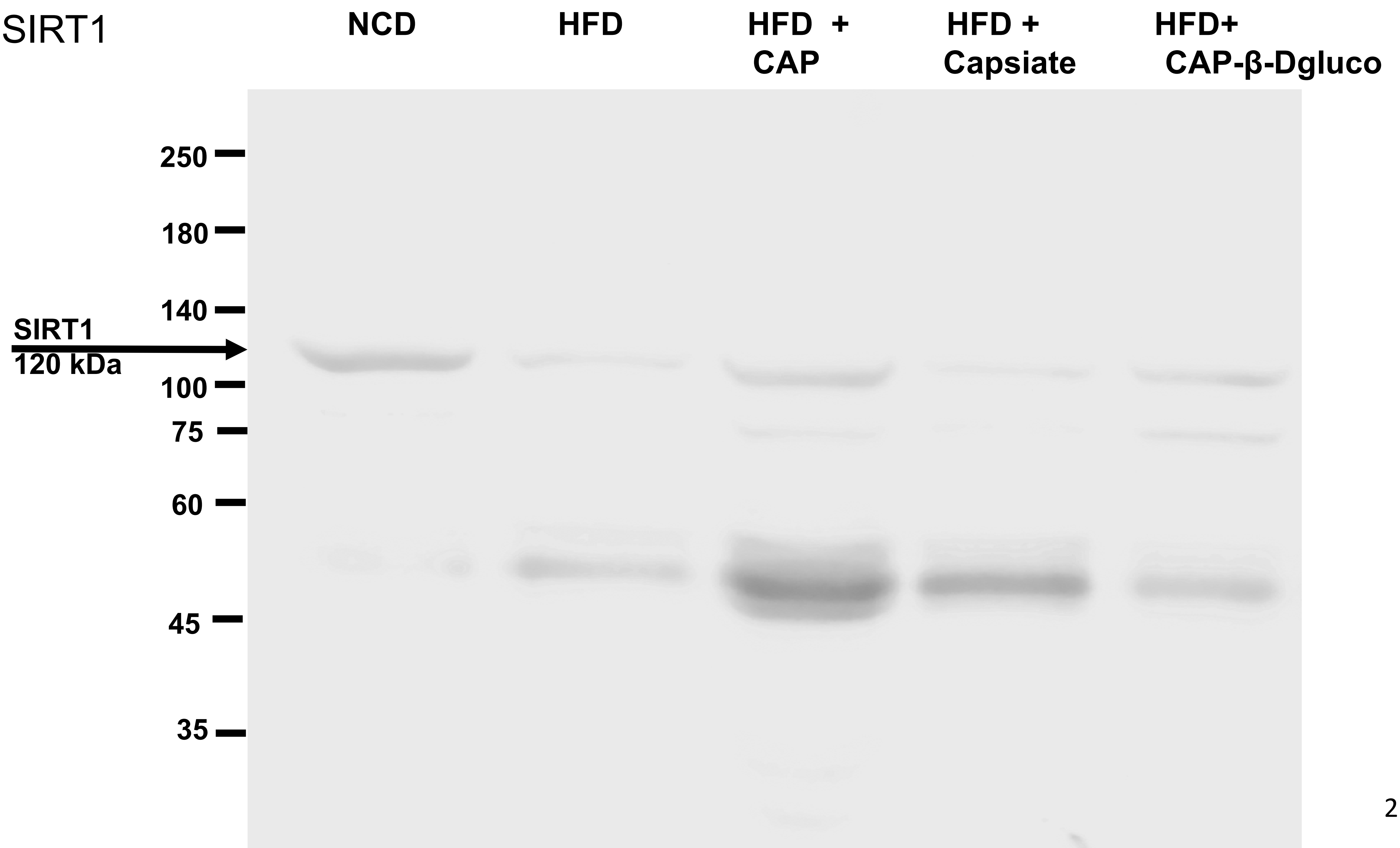
NCD
HFD
HFD +
CAP
HFD +
Capsiate
HFD+
CAP-β-Dgluco
250
180
140
SIRT1
120 kDa
100
75
60
45
35

SIRT1

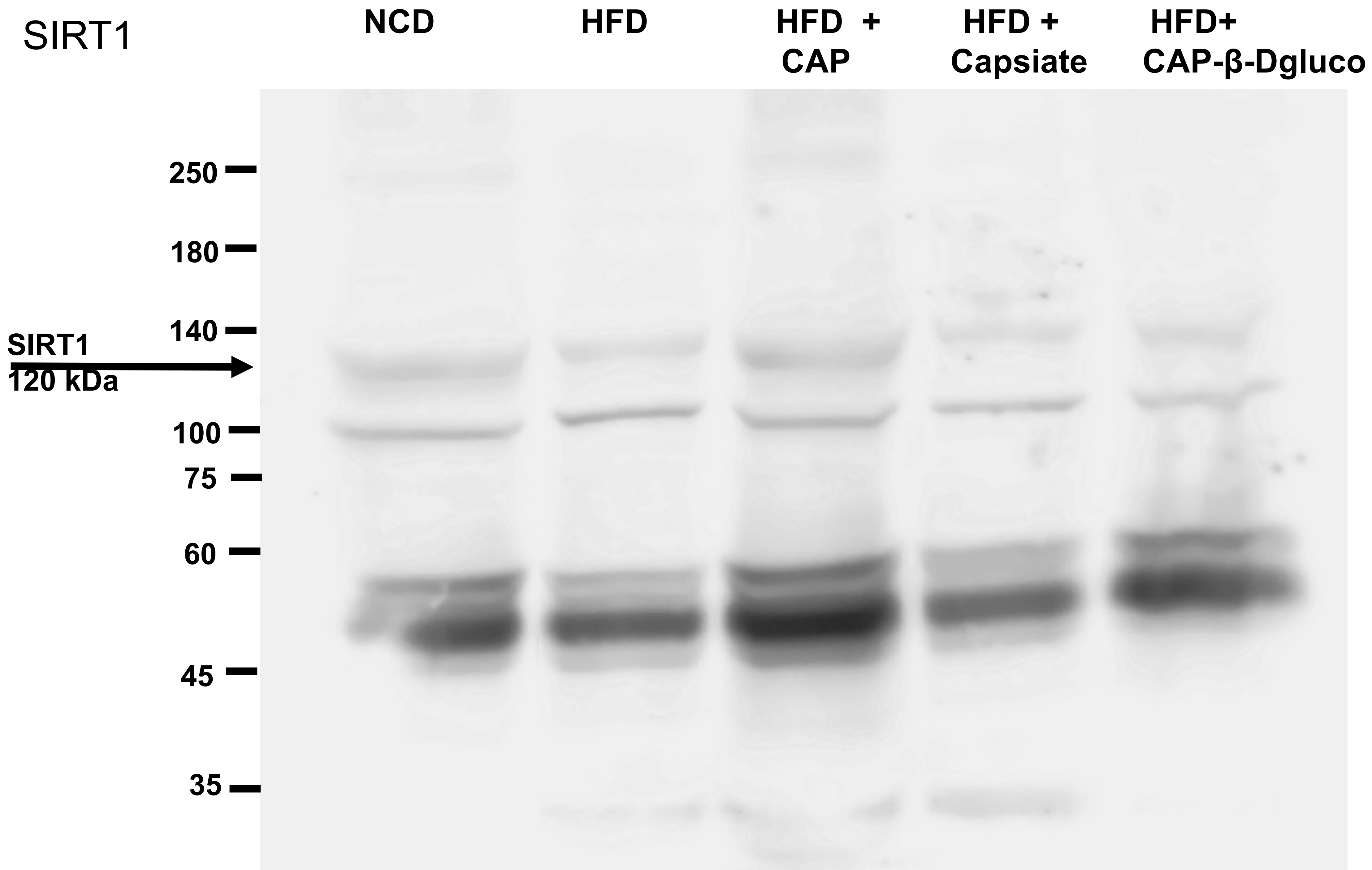
NCD
HFD
HFD + CAP
HFD + Capsiate
HFD+ CAP-β-Dgluco
250
180
140
SIRT1 120 kDa
100
75
60
45
35

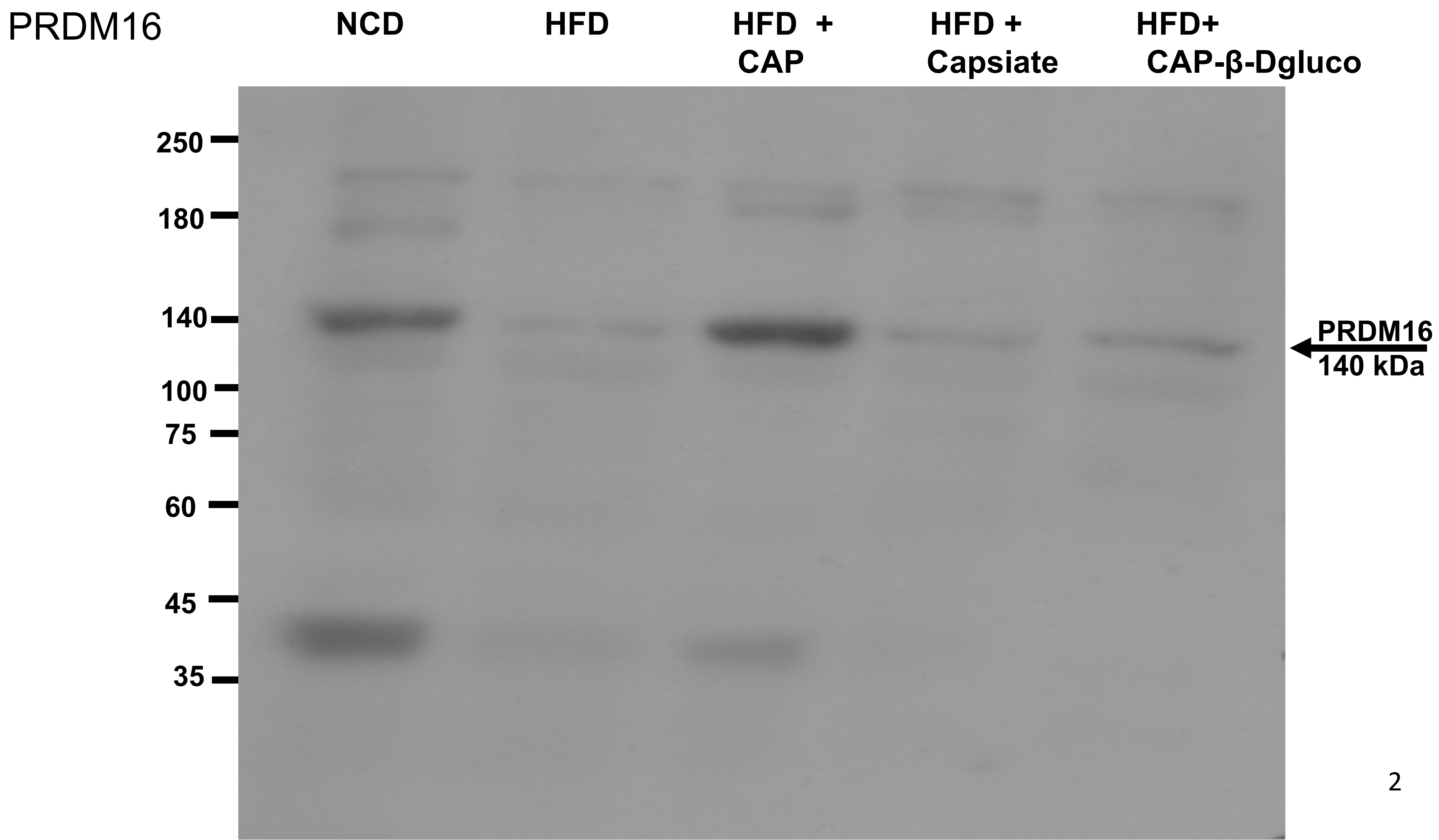
PRDM16
NCD
HFD
HFD + CAP
HFD + Capsiate
HFD+ CAP-β-Dgluco
250
180
140
100
75
60
45
35
PRDM16
140 kDa
2

PRDM16

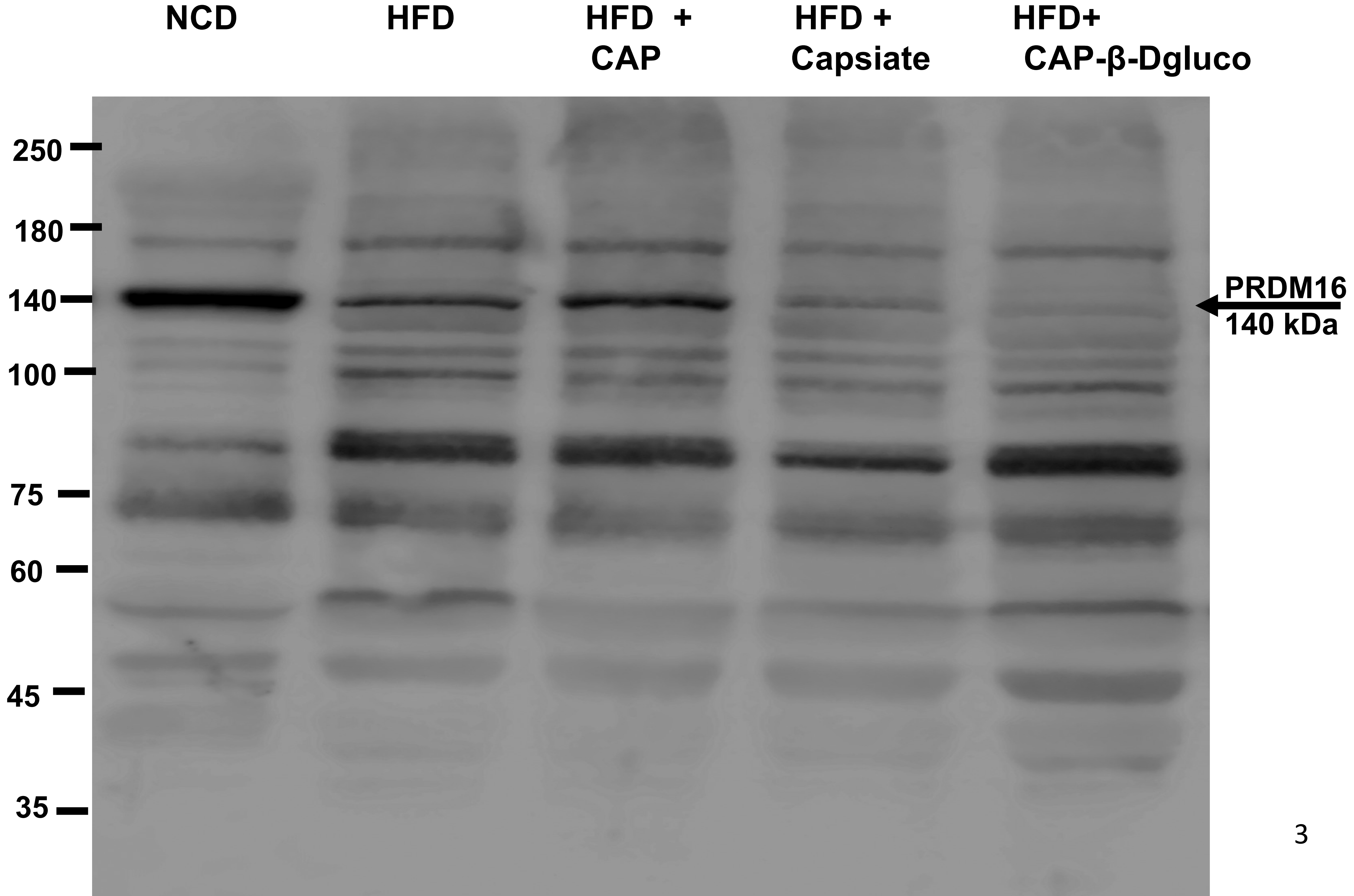
NCD
HFD
HFD + CAP
HFD + Capsiate
HFD+ CAP-β-Dgluco
250
180
140
100
75
60
45
35
PRDM16
140 kDa

PGC1α

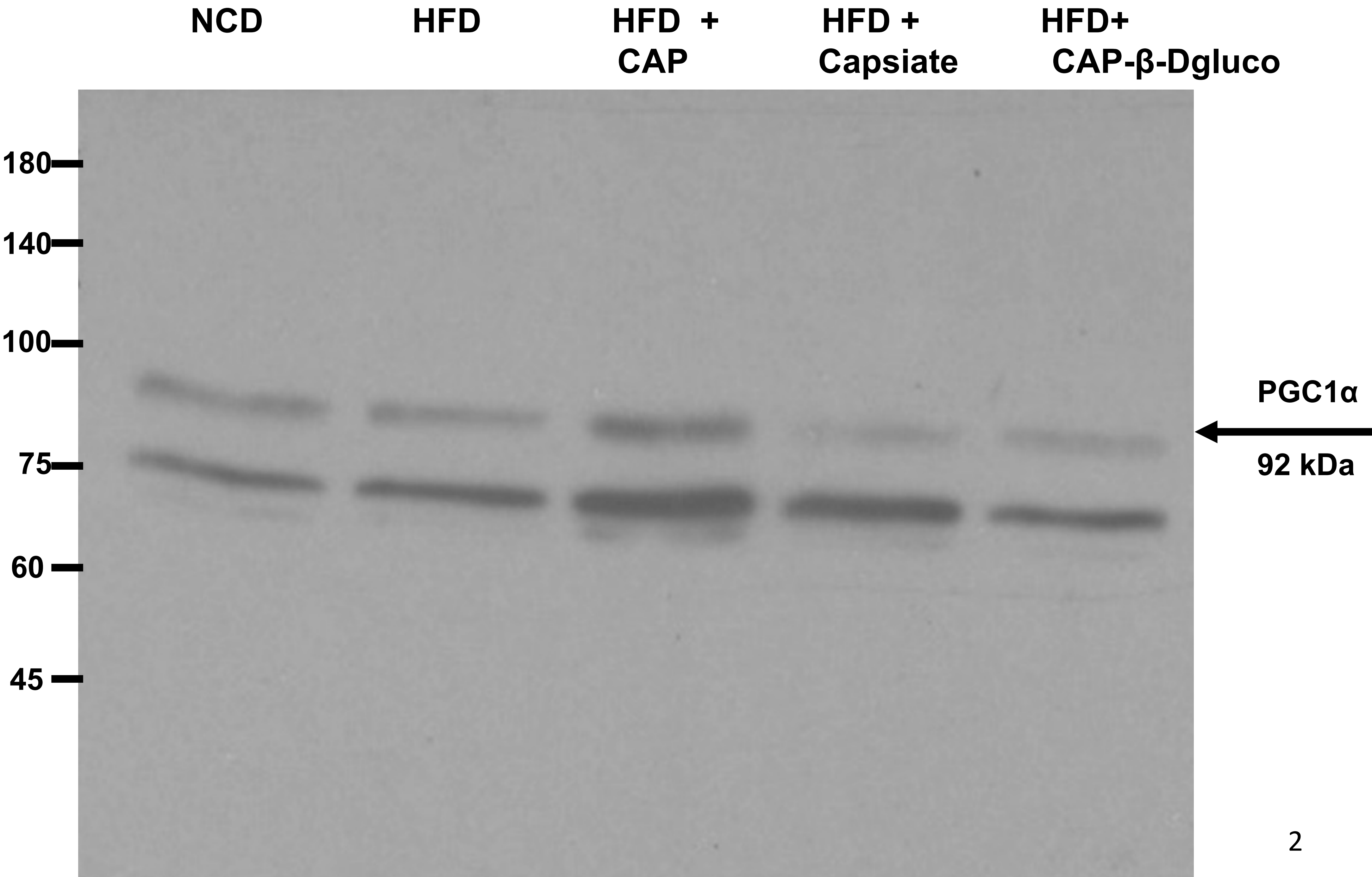
NCD
HFD
HFD +
CAP
HFD +
Capsiate
HFD+
CAP-β-Dgluco
180
140
100
75
60
45
PGC1α
92 kDa

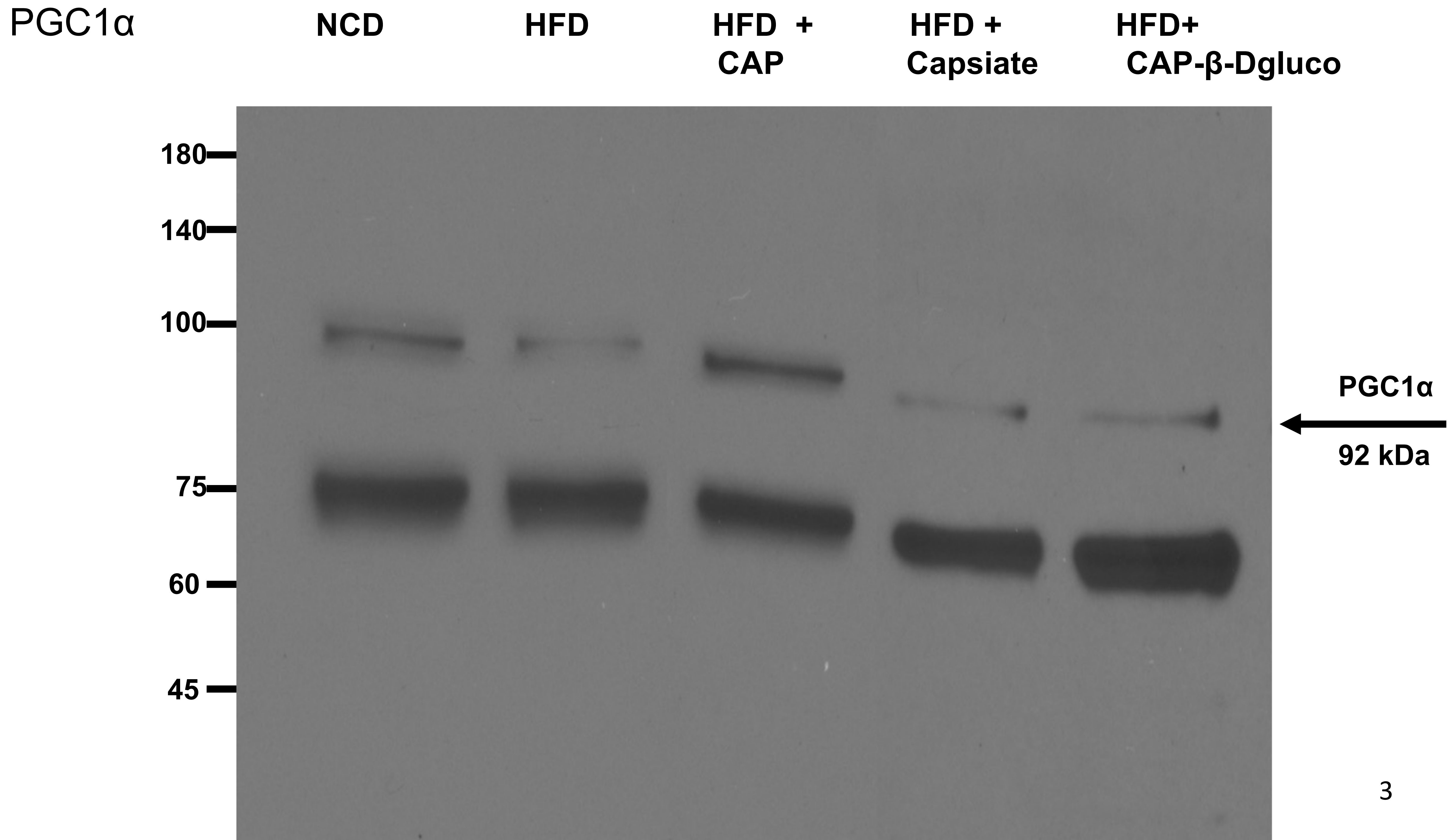
PGC1α
NCD
HFD
HFD + CAP
HFD + Capsiate
HFD+ CAP-β-Dgluco
180
140
100
75
60
45
PGC1α
92 kDa

GAPDH

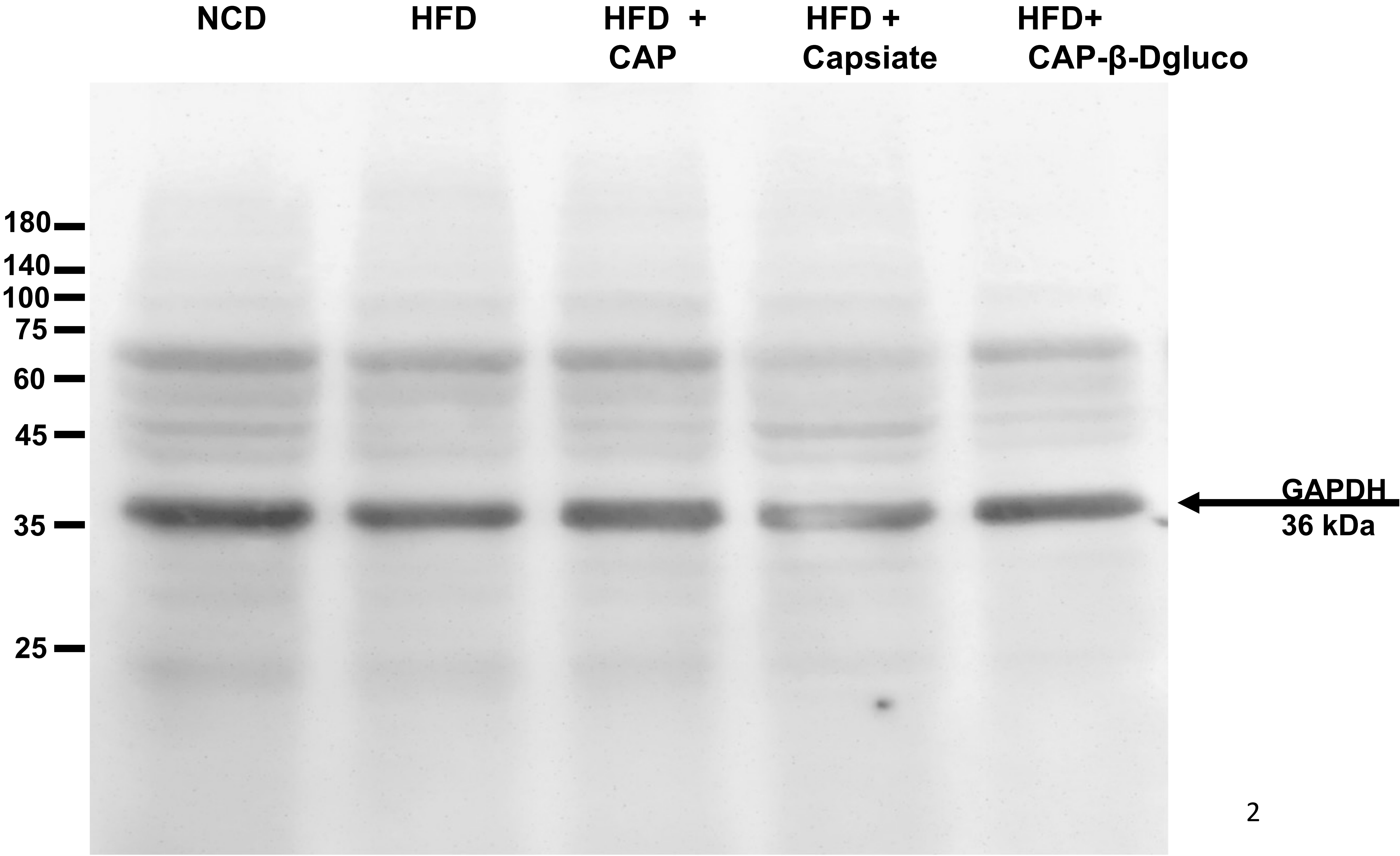
NCD
HFD
HFD + CAP
HFD + Capsiate
HFD+ CAP-β-Dgluco
180
140
100
75
60
45
35
25
GAPDH
36 kDa

GAPDH

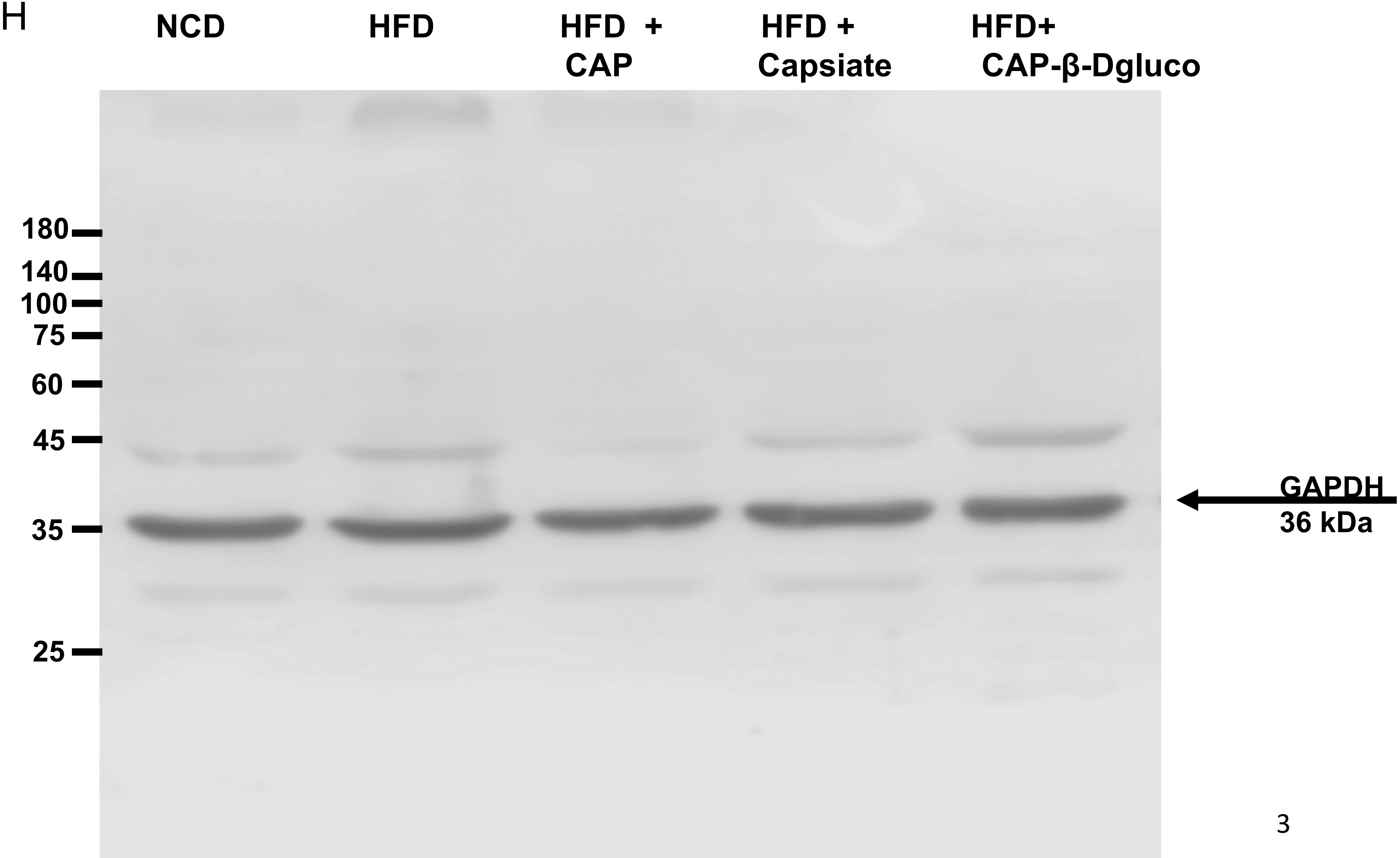
NCD
HFD
HFD + CAP
HFD + Capsiate
HFD+ CAP-β-Dgluco
180
140
100
75
60
45
35
25
GAPDH
36 kDa
