# Supplementary figures and images for: Binding Efficacy and Thermogenic Efficiency of Pungent and Nonpungent Analogs of Capsaicin
Source: Molecules. 2018 Dec 4;23(12):3198. doi: 10.3390/molecules23123198 (PMC6321193; doi:10.3390/molecules23123198)

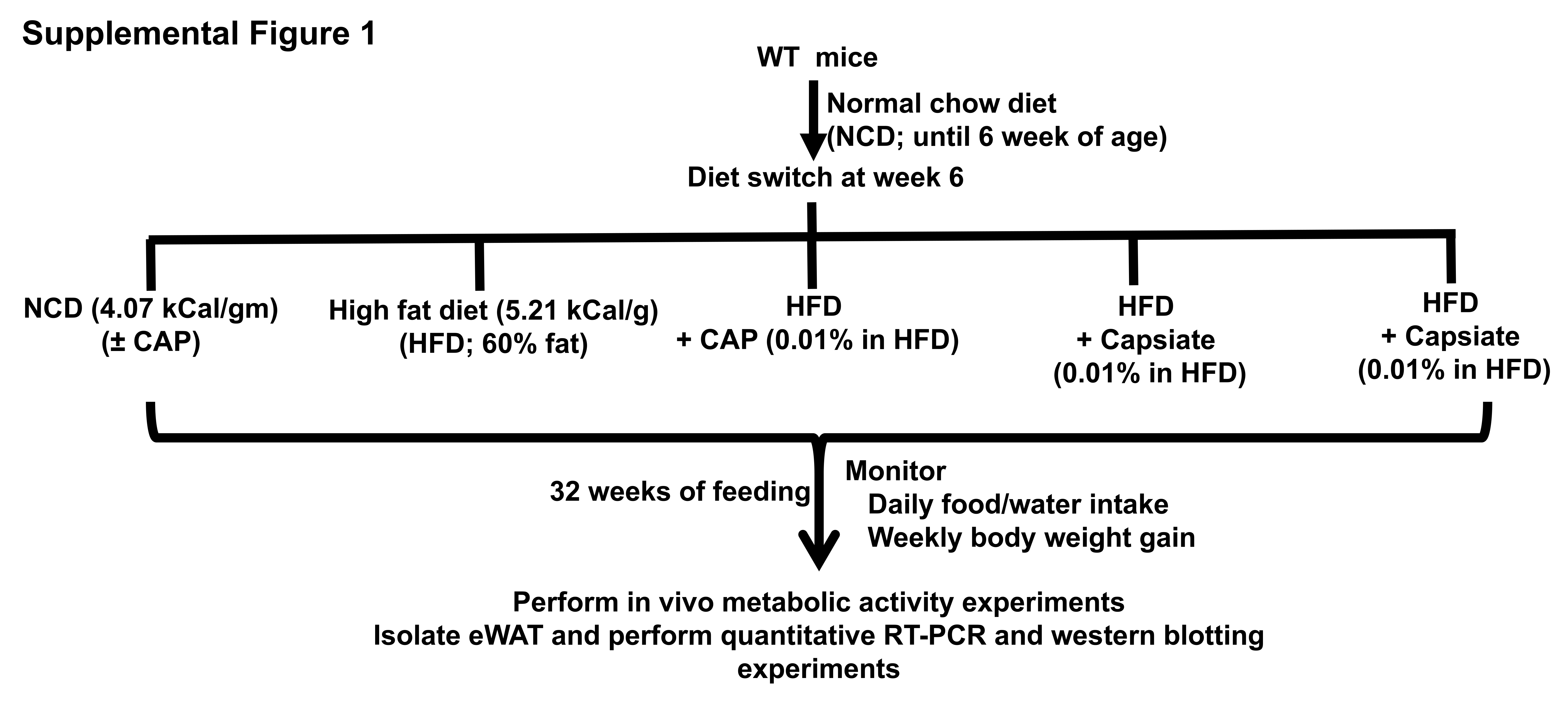

Supplement: Supplementary file 1 [file molecules-23-03198-s001.zip › Supplemental Figure 1.jpg]

TRPV1

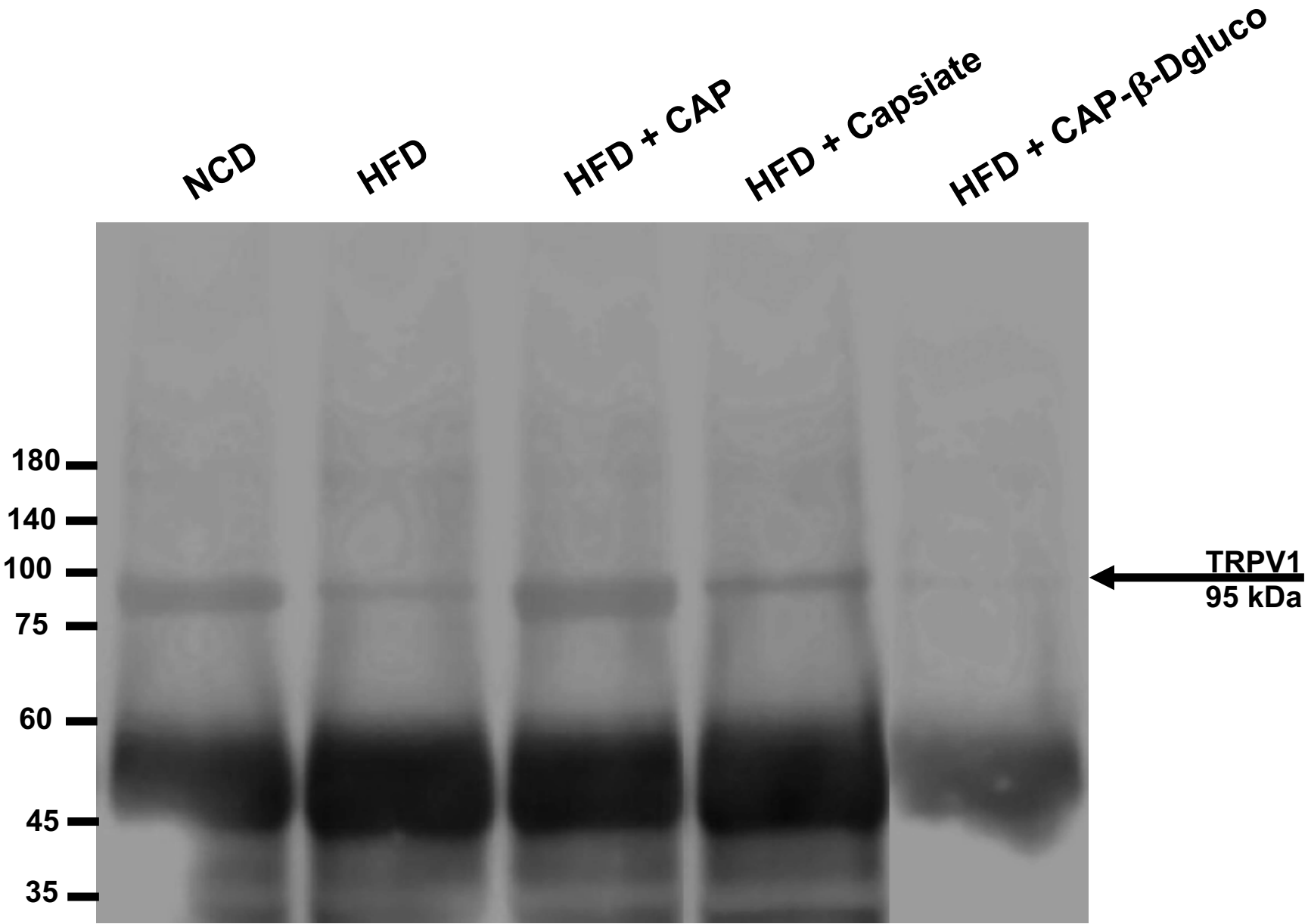

PPAR $\alpha$

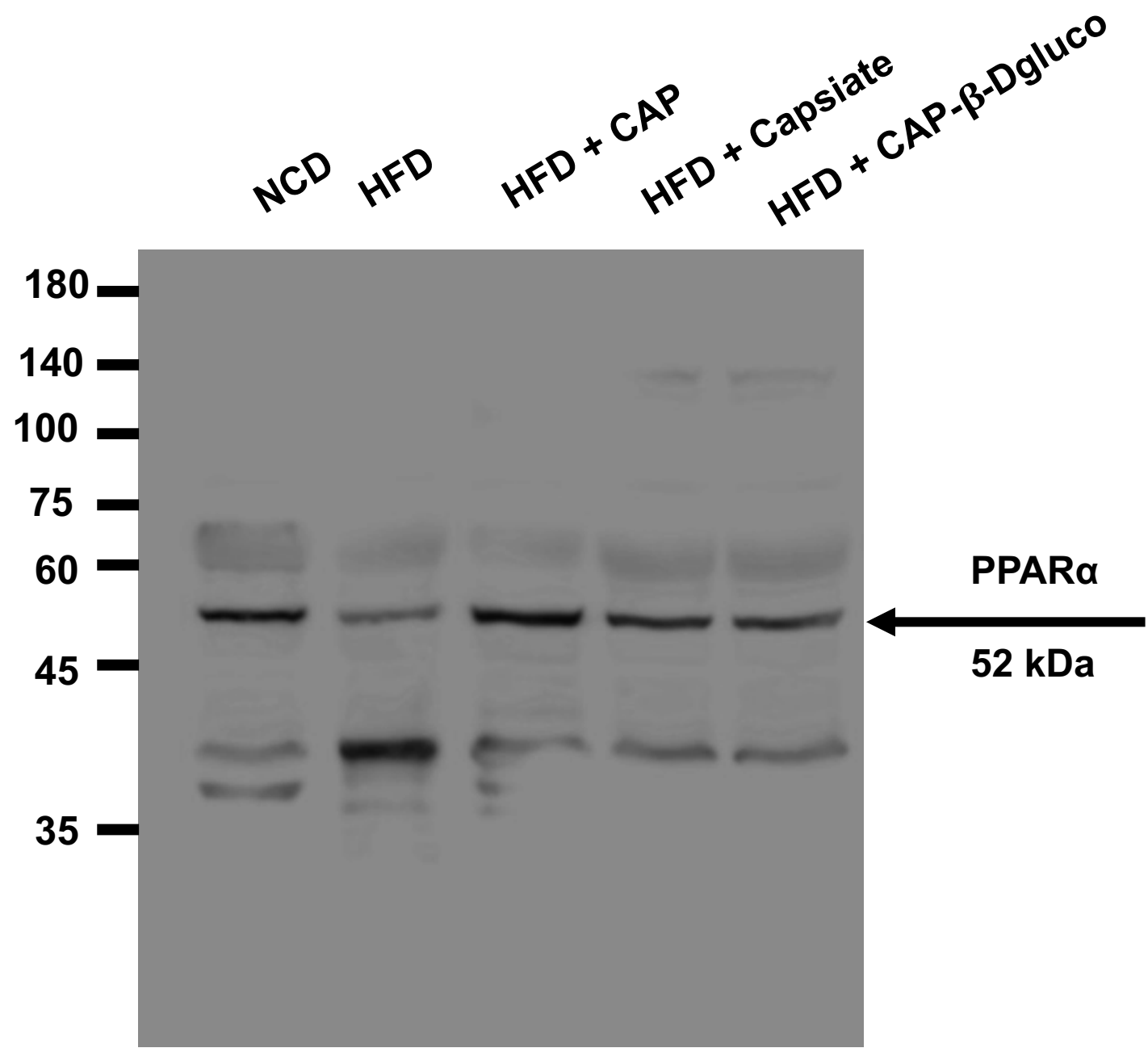

# BMP8b

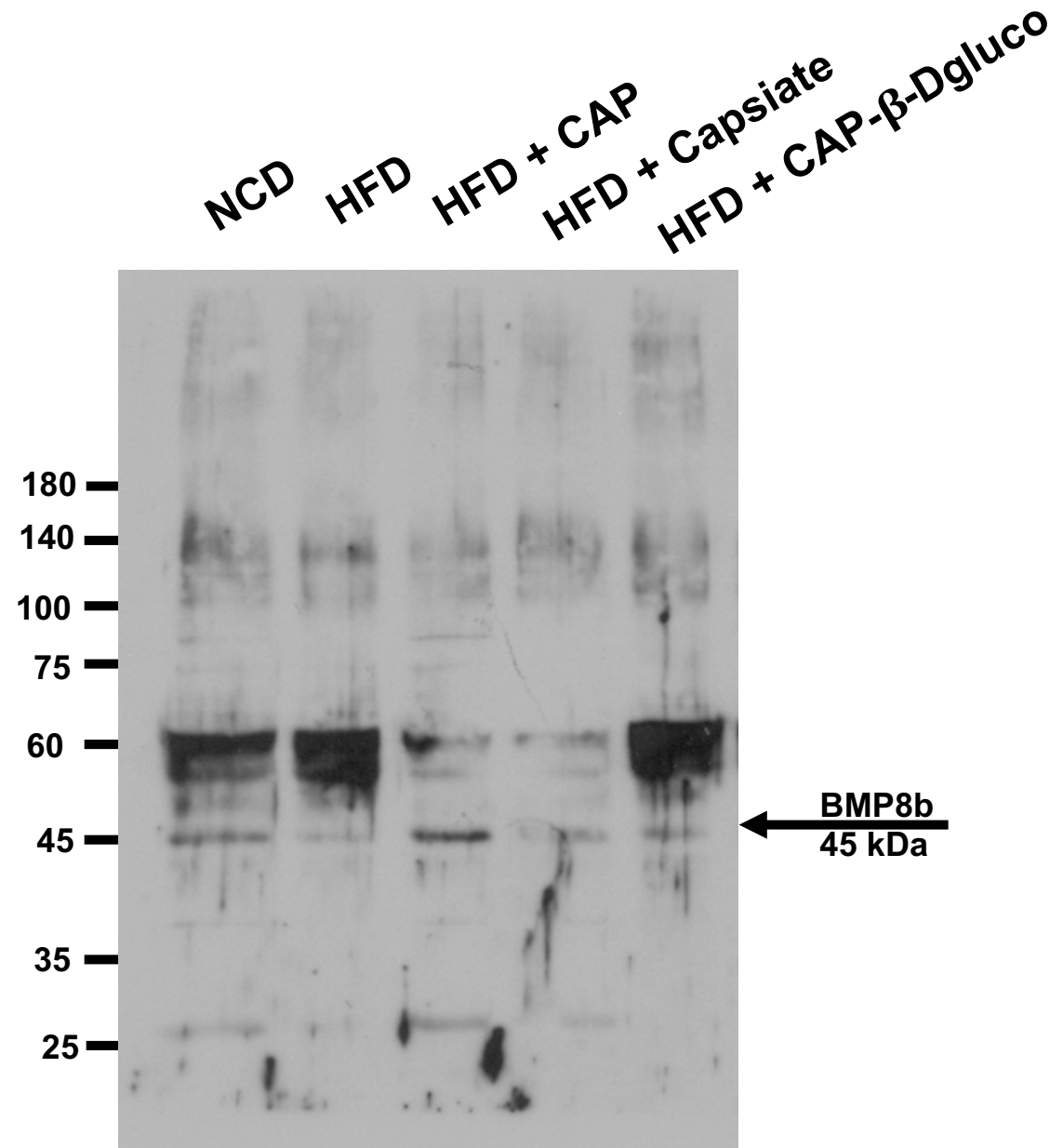

# UCP-1

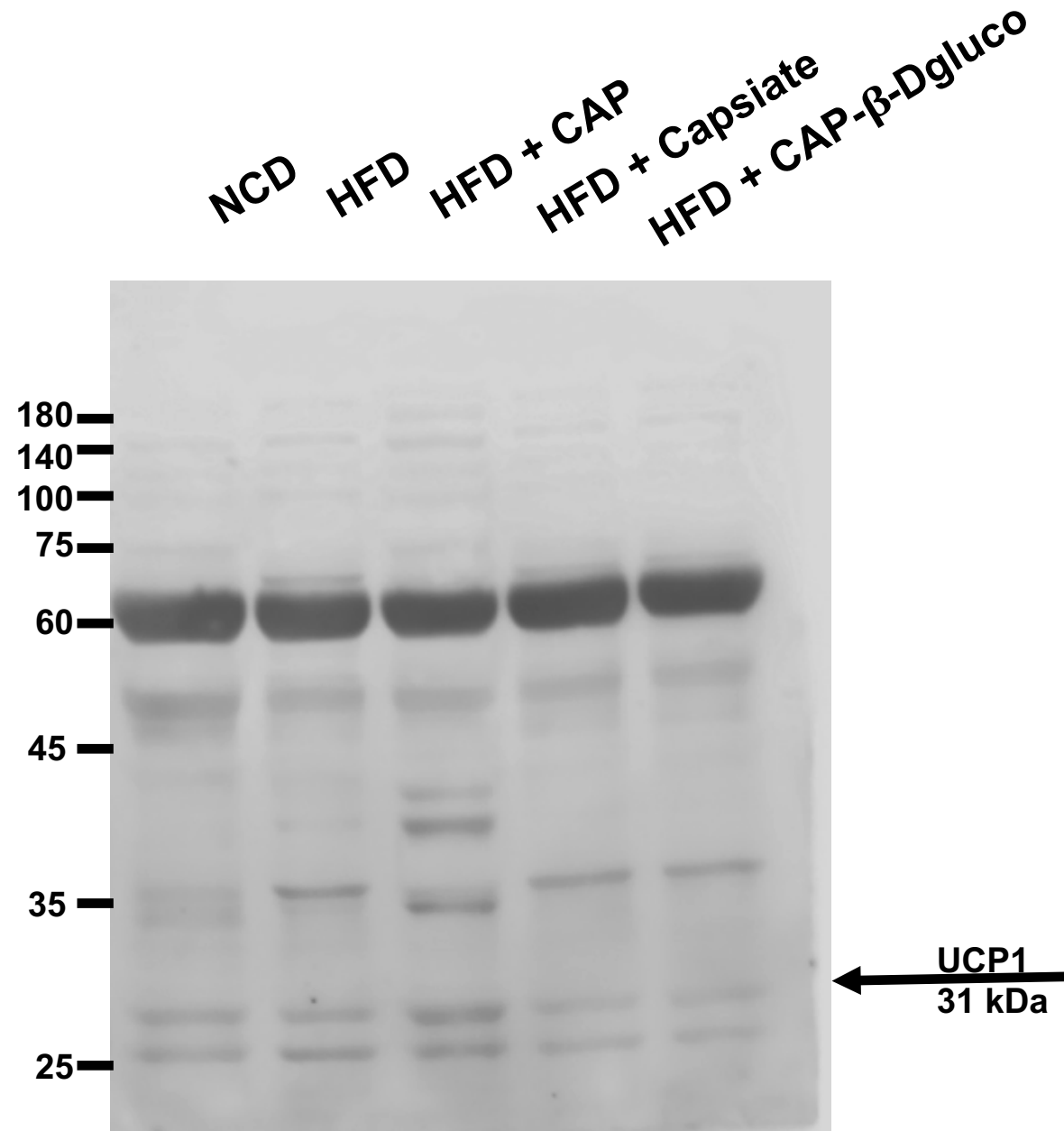

# SiRT-1

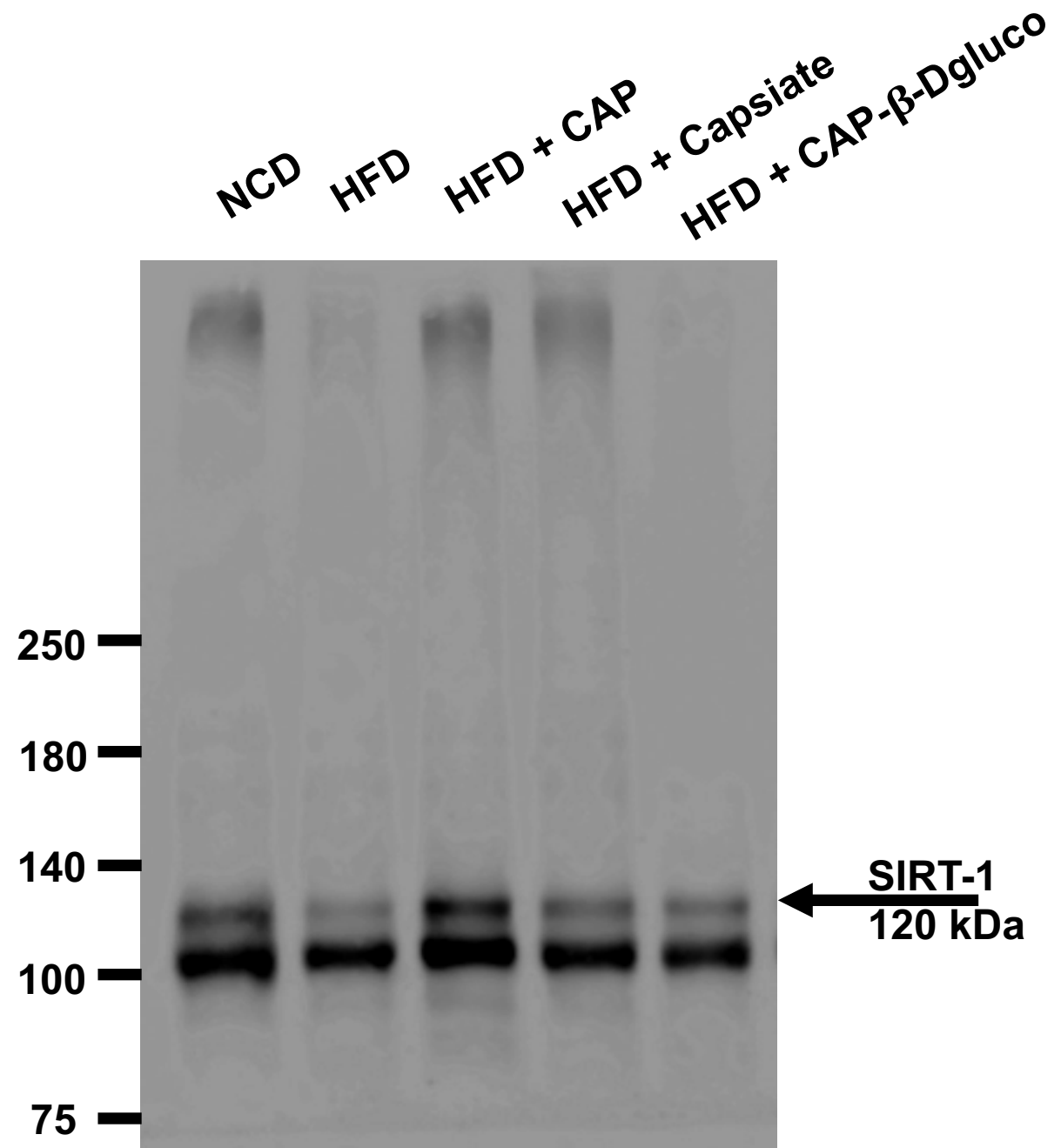

# PGC-1 $\alpha$

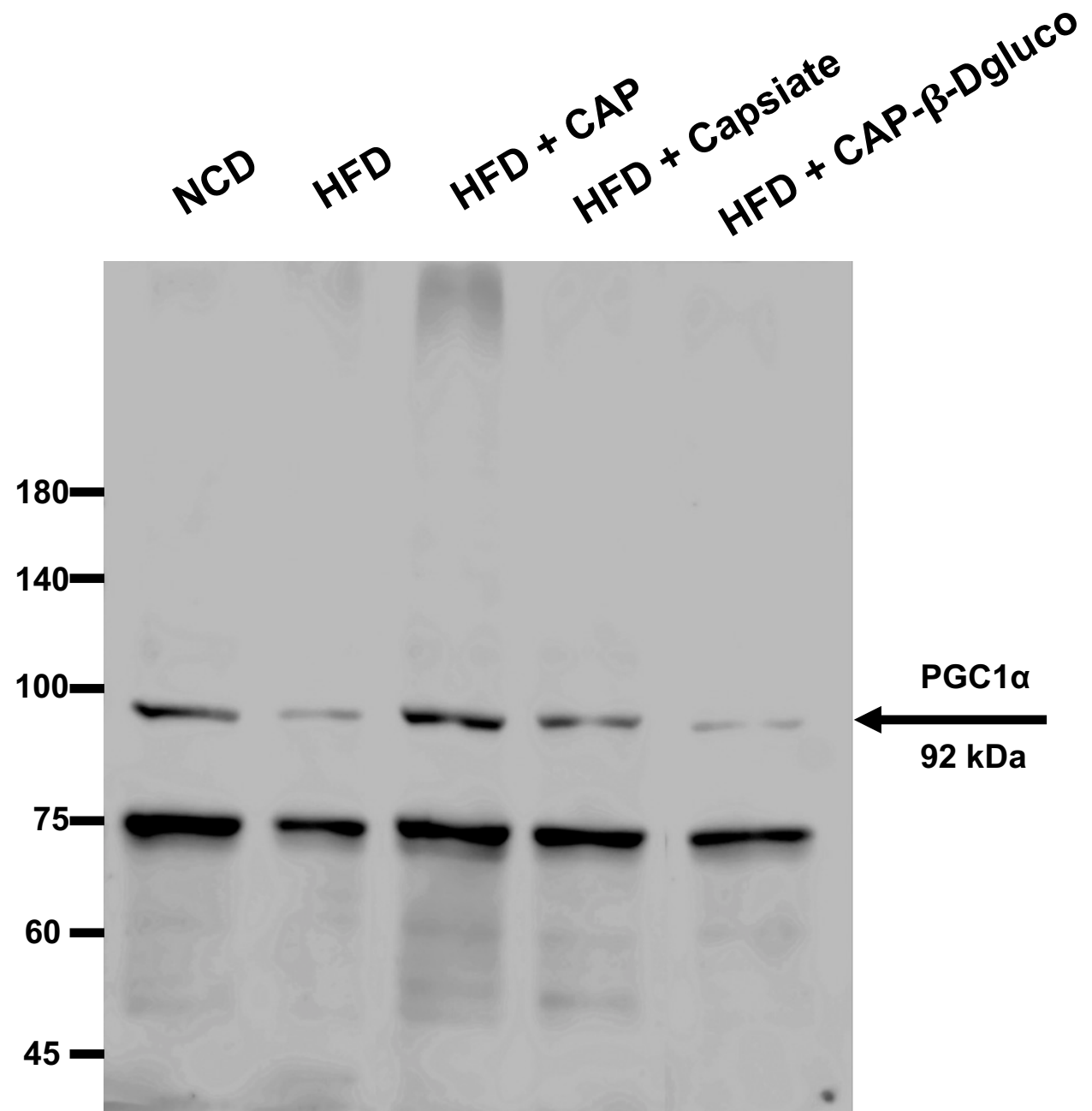

PRDM-16

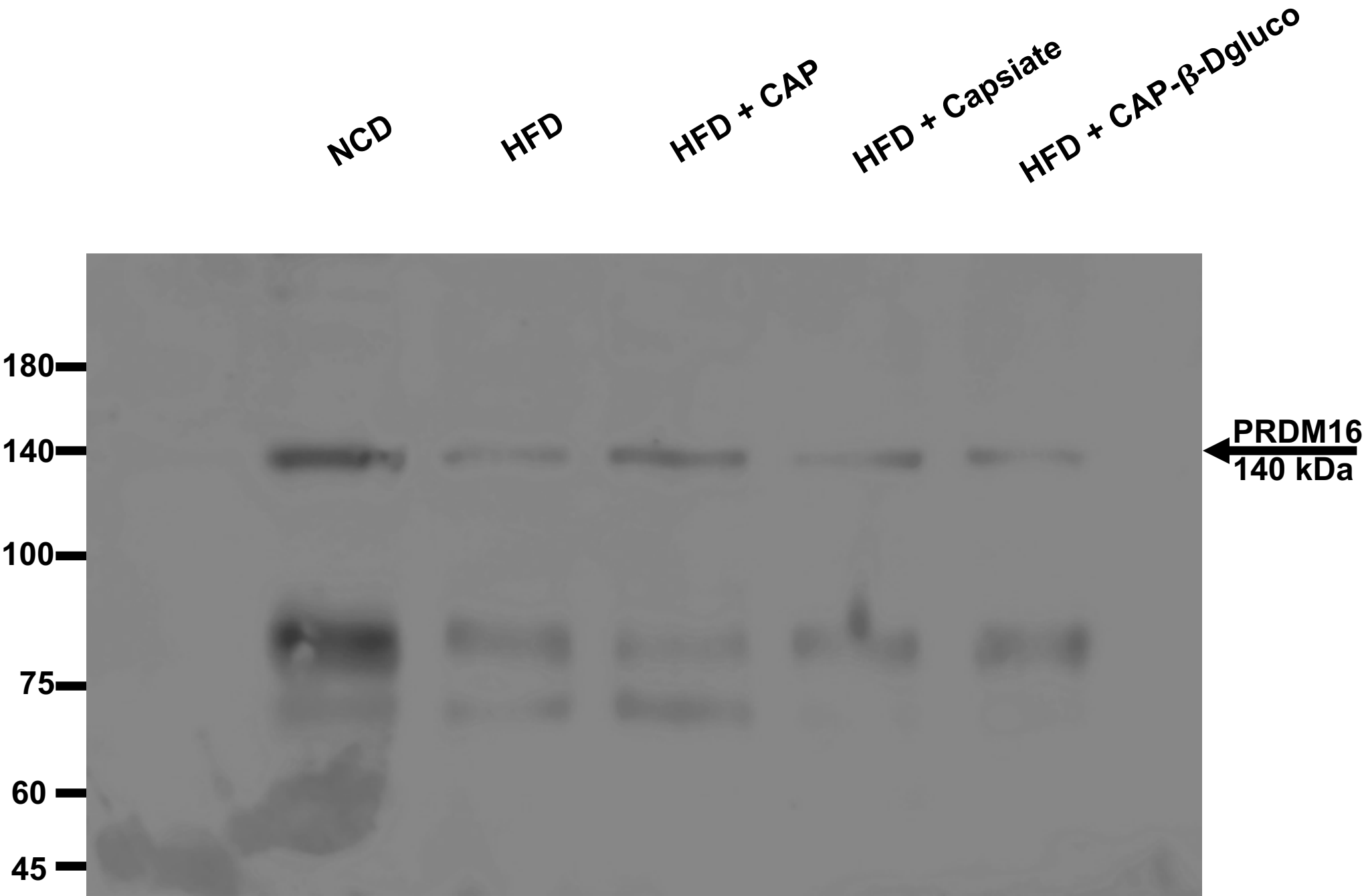

# GAPDH

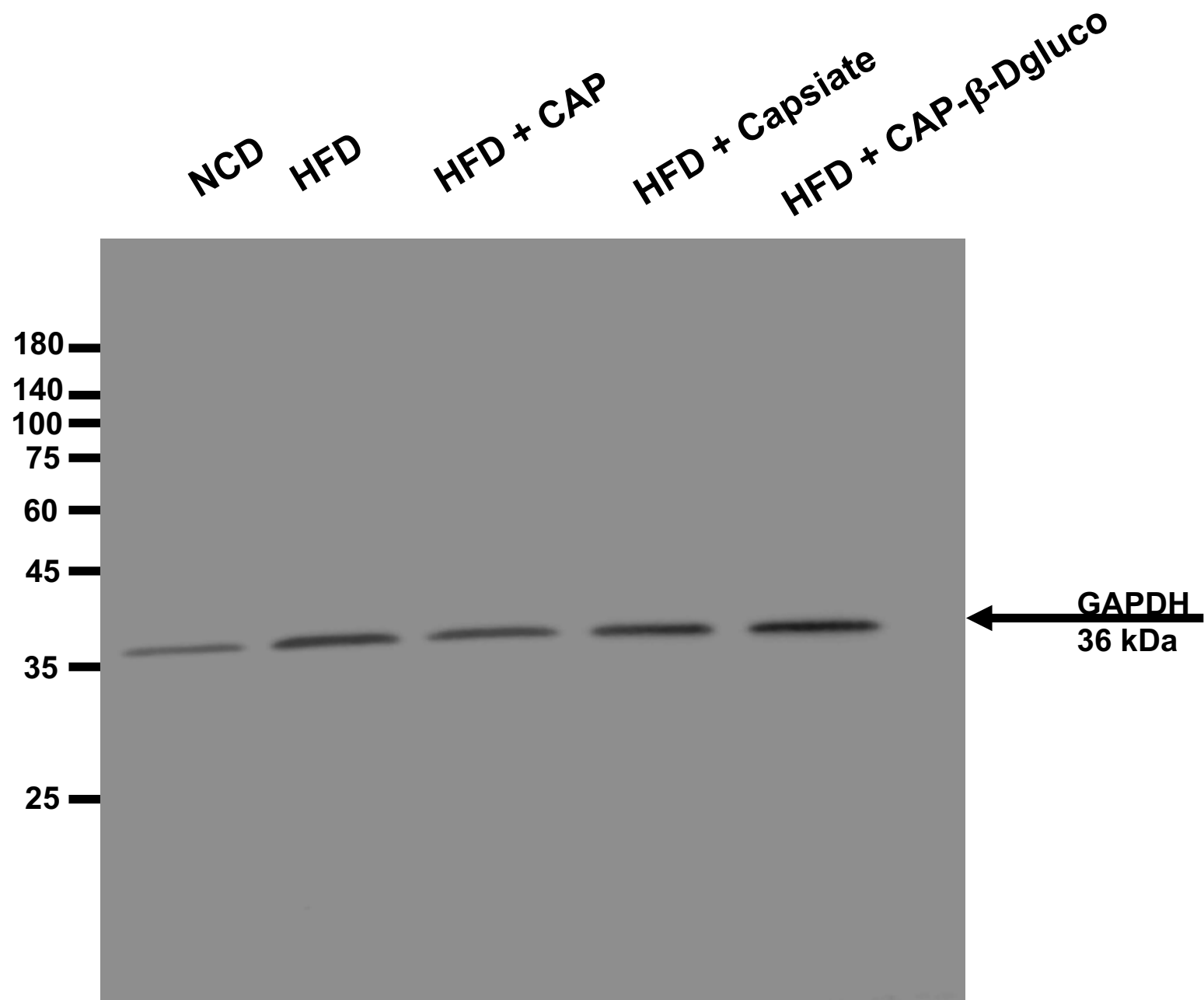

Supplement: Supplementary file 1 [file molecules-23-03198-s001.zip › Supplemental Figure 2.pdf]
